# Dengue Spatial and Temporal Patterns, French Guiana, 2001

**DOI:** 10.3201/eid1004.030186

**Published:** 2004-04

**Authors:** Annelise Tran, Xavier Deparis, Philippe Dussart, Jacques Morvan, Patrick Rabarison, Franck Remy, Laurent Polidori, Jacques Gardon

**Affiliations:** * Institut de Recherche pour le Développement Guyane, Cayenne, Guyane; † Institut Pasteur de la Guyane, Cayenne, Guyane; ‡ Centre Hospitalier de Cayenne,Cayenne, Guyane

**Keywords:** Epidemic, dengue fever, Geographic Information System, space-time analysis, control strategy

## Abstract

To study a 2001 dengue fever outbreak in Iracoubo, French Guiana, we recorded the location of all patients’ homes and the date when symptoms were first observed. A geographic information system was used to integrate the patient-related information. The Knox test, a classic space-time analysis technique, was used to detect spatiotemporal clustering. Analysis of the relative-risk (RR) variations when space and time distances vary, highlighted the maximum space and time extent of a dengue transmission focus. The results show that heterogeneity in the RR variations in space and time corresponds to known entomologic and epidemiologic factors, such as the mosquito feeding cycle and host-seeking behavior. This finding demonstrates the relevance and potential of the use of GIS and spatial statistics for elaborating a dengue fever surveillance strategy.

While investigating the spatial patterning of health events and disease outcomes has a long history (*1*), the development of geographic information systems (GIS) has recently enabled epidemiologists to include a spatial component in epidemiologic studies more easily. GIS are computer systems that allow the collection, storage, integration, analysis, and display of spatially referenced data. In the field of health, GIS have been widely used for disease mapping of different pathologies, in analysis of space and space-time distributions of disease data (*2**–**5*), in identifying risk factors (*6**–**8*), and in mapping risk areas (*9*). In most studies, each patient or person exposed to a disease is located at the residential address, and these locations are integrated into a GIS for mapping and analysis. Because GIS allows epidemiologists to map environmental factors associated with disease vectors, it has become especially relevant for the surveillance of infectious and vector-borne diseases such as malaria (*3**,**8**,**10*) or Lyme disease (*11**–**13*).

In particular, GIS and spatial statistics should be useful for surveillance of dengue fever (DF), an arboviral disease transmitted to humans by mosquitoes of the *Aedes* genus (*14*). Indeed, because no vaccine or specific treatment is available, the only solution to prevent the disease is vector control strategy. This control strategy requires that risk areas and risk periods be identified. Several studies, some in which GIS was used, have been conducted to identify the mechanisms of the spread of dengue viruses in a community and to improve prevention strategies (*4**,**15**–**17*). The existence of case-clusters inside the same house has often been described (*4**,**15**,**16**,**18**–**24*). Moreover, by a space-time analysis of reported dengue cases in Puerto Rico, Morrison et al. have shown the apparent clustering of cases at short distances over brief periods of time (*4*). Nevertheless, limits of this cluster have not been calculated.

To better understand the transmission dynamics of dengue, we used a GIS to describe the spread of dengue viruses in a small locality. Data were obtained from a recent dengue fever outbreak in Iracoubo, a small town located in French Guiana, an overseas French administrative unit between Suriname and North Brazil.

In French Guiana, DF is recognized as endemic, with dengue epidemics occurring since 1965 at 4- to 6-year intervals (*25*). The four dengue virus serotypes (DEN-1, DEN-2, DEN-4, and more recently DEN-3) have been isolated. The mosquito *Aedes aegypti* is the only known dengue vector in French Guiana. We report the investigation of space-time patterns of confirmed laboratory-positive and suspected cases; evaluate the efficiency of using GIS technologies in a dengue prevention program, and propose a surveillance strategy.

## Materials and Methods

### Study Site and Population

**Figure 1:**
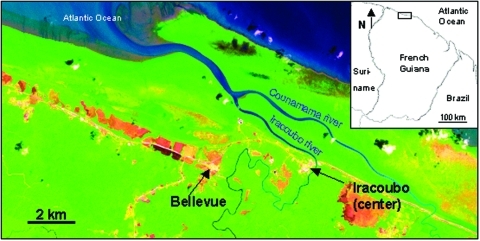
Iracoubo, French Guiana (Landsat TM imagery). Housing areas (pink) are surrounded by coastal wetlands (orange), rain forest and mangrove forests (green areas). Ocean and rivers are in blue.

Iracoubo is a small rural municipality located on the coastal plain of French Guiana with a population of 1,428 inhabitants (*26*), most of whom live in the main town or in the Bellevue village, located 5 km from the main town (Figure 1). Housing areas are surrounded by rain forest, mangrove forest, and coastal wetlands.

### Patients

All patients who visited the healthcare center of Iracoubo with a temperature of >38.5°C, arthralgia, headache, or myalgia, were suspected of having DF. Blood samples were taken for evaluation of probable and confirmed DF cases. The terms suspected, probable, and confirmed cases of DF were used according to the definitions adopted by the Council of State and Territorial Epidemiologists and the Centers for Disease Control and Prevention (CDC), Atlanta, Georgia (*27*). A suspected case is defined as an illness in a patient whose serum was sent to National Reference Centre for Arboviruses (Institut Pasteur de la Guyane, Cayenne, French Guiana) for the diagnosis of DF. A probable case was an illness in a person that is clinically compatible with dengue, combined with supportive serologic test results (a single convalescent-phase serum specimen containing dengue virus immunoglobulin [Ig] M antibody, or a dengue virus IgG antibody titer of >1,280 by hemagglutination inhibition assay [HI]). A confirmed case was defined as having any of the following criteria: isolation of dengue virus from serum, demonstration of a dengue virus cDNA fragment by amplification (reverse transcription–polymerase chain reaction [RT-PCR]) from a serum sample, IgM antibody seroconversion, or a fourfold or greater increase in reciprocal titers of IgG antibody to one or more dengue virus antigens in paired serum samples.

During a dengue epidemic in a disease-endemic area such as French Guiana, the predictive positive value for a probable dengue case to be a confirmed case is very high (24). For this reason, we decided to include the probable dengue cases in the group of confirmed cases. Thus, we use the term confirmed case for both probable and confirmed dengue cases, and the term suspected case for all reported cases during the epidemic.

### Laboratory Diagnosis

All tests were performed at the Institut Pasteur de la Guyane, National Reference Centre for Arboviruses (Cayenne, French Guiana).

### Serologic Tests

Two techniques were used to detect antibodies to dengue viruses. The first was detection of IgM dengue virus antibodies by using an IgM capture enzyme-linked immunosorbent assay (MAC-ELISA) with a tetravalent dengue virus antigen. The procedure was modified from a previously described method (*28*).

HI was also used. HI titers were determined by using the method of Clarke and Casals (*29*) that was adapted to a microtechnique. Antibody responses to dengue virus were interpreted according to the World Health Organization criteria (*30*).

### Virus Isolation and Identification

Acute-phase serum samples from febrile patients (<4 days after the onset of fever) were diluted 10-fold in Leibowitz medium containing 3% fetal calf serum, and dilutions were injected into subconfluent AP 61 cell cultures as previously described (*31*). After 7 days of culture, cells were harvested, and dengue viruses were identified according to serotype by an indirect immunofluorescence assay (IFA) with monoclonal antibodies specific to DEN-1, -2, -3, and -4 viruses (provided by CDC, Fort Collins, CO).

### Detection of Dengue Viruses by Using RT-PCR Analysis

Viral RNA was extracted from a 50-μL aliquot of acute-phase serum with TRIzol (Invitrogen Life Technologies, Paisley, Refrewshire, UK), according to the manufacturer’s recommendations and precipitated with isopropanol and 1 μL of glycogen (5 μg/μL) (Roche Diagnostics, Mannheim, Germany). Air-dried RNA pellets were suspended in 20 μL of water. Then, 5 μL of RNA were mixed with 200 ng of random hexamer primers, and first-strand cDNA synthesis was performed with the SuperScript First-Strand Synthesis System for RT-PCR (Invitrogen Life Technologies), according to the manufacturer’s recommendations. The first run of RT-PCR analysis and subsequent seminested PCR analysis were performed following a previously described procedure (*32*).

### Cases Georeferencing

For all suspected dengue patients, patient’s home was recorded with a cadastral map (paper copy, scale 1/1,000). Georeferenced aerial photographs were used to improve the identification and the location. The geographic coordinates were integrated into a GIS (Geoconcept software) (*33*), with the following information about the patient: identification number, date of onset of symptoms, age, sex, diagnosis.

### Spatial and Temporal Patterns Analysis

Assuming that DF spread within a community leads to the creation of transmission focus, the distance between neighboring housing would be an important factor in the spatial extension of these foci. We used the GIS and geocoded aerial photographs to locate all houses and to calculate the mean distance between neighboring houses (Geoconcept software) (*33*).

We used the Knox (*34*) test to identify possible space-time interactions, i.e., to determine whether cases which are close in distance will also be close in time. This method evaluates whether the number of pairs of cases found at a fixed temporal and spatial distances is substantially different from the number of pairs of cases expected at these distances by chance, when the times of occurrence of cases are randomly distributed across the case locations.

The ratio between real number of pairs of cases found at the space-distance *s* (in meters) and the time-distance *t* (in days) and the number of pairs of cases found at these distances by chance could be considered as the RR of occurrence of another dengue case, *t* days later and *s* meters away from the first case of dengue.

The Knox test was first computed for the population of patients with confirmed cases and for the population of those with suspected cases. Results were calculated for time distances varying from 1 to 200 days (duration of the epidemic) by 1-day step and spatial distances varying from 5 to 6,500 m (step: 5 m). An “RR map” was then obtained by interpolating the significant values (p = 0.05) (Surfer software) (*35*).

The final result is a representation of the RR, when space-distance and time-distance from a hypothetical dengue patient vary. The correlation between RR values derived from the confirmed cases and those derived from the suspected cases was evaluated.

## Results

### Serologic Tests

In Iracoubo center and Bellevue, 161 patients with suspected dengue cases were reported between April and November 2001, which corresponds to 11.3% of the population. Blood samples from 57 patients were analyzed in the National Reference Center for Arboviruses, Arbovirology Laboratory of the Pasteur Institute of French Guiana. Among the 57 patients, 32 cases of DF were confirmed (56.1%). A total of 25 suspected cases were not confirmed; among them 4 cases were indeterminate and 21 were negative (Table). Virus isolation results show that the majority of confirmed cases were caused by DEN-3 (90%).

**Table:** Serologic analysis results

	Iracoubo center	Bellevue	Total
Reported cases	93	69	162
Analyzed	34	23	57
Laboratory-negative	14	7	21
Indeterminate	1	3	4
Laboratory-positive	19	13	32
Confirmed	14	8	22^a^
Probable	5	5	10

^a^19 DEN-3; 2 DEN-1, 1 seroconversion.

### Epidemic Description

**Figure 2:**
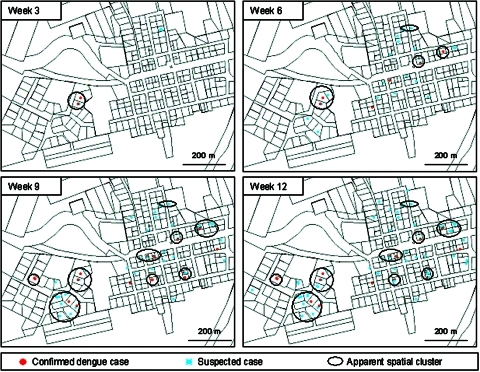
Maps showing locations of dengue patients in Iracoubo center (weeks 3, 6, 9, 12), showing locations of confirmed (red) and suspected (blue) dengue fever patients. Black circles correspond to spatial clusters, that is, neighborhoods where more than two confirmed cases or three suspected cases occurred.

**Figure 3:**
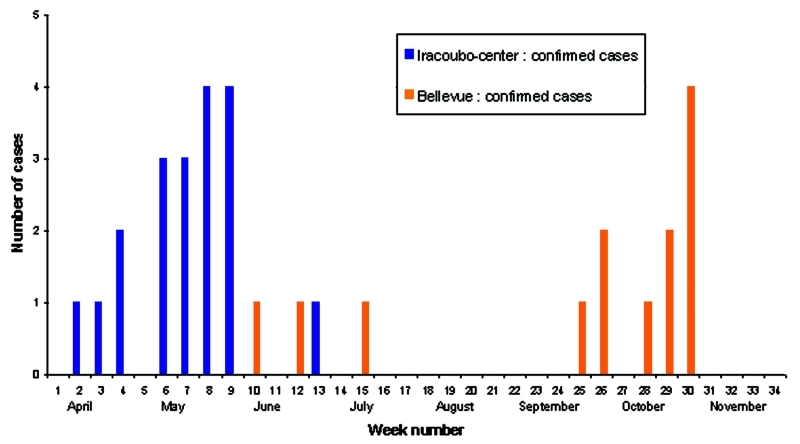
Number of confirmed dengue fever cases per week, Iracoubo municipality, French Guiana, April–November 2001.

The first suspected dengue case was reported on April 10 but samples were not analyzed. The first confirmed dengue case occurred on April 22. Then, the epidemic spread rapidly through the community (Figure 2), with a temporal lag between cases occurring in Iracoubo center and those occurring in Bellevue (Figure 3). Indeed, 100% of the confirmed cases in Iracoubo center occurred between April and July, whereas 76.9% of confirmed cases in Bellevue occurred in October and November. The first glimpse of the spatial distribution of confirmed and suspected cases shows the existence of apparent spatial clusters (more than 2 confirmed cases or 3 suspected cases in the same neighborhood) (Figure 2).

### Spatial-Temporal Patterns Analysis

We considered 406 buildings in the calculation. The mean distance between adjacent houses was 24.6 m for the whole municipality, including means of 22.6 m for Iracoubo center and 29.6 m for Bellevue.

**Figure 4:**
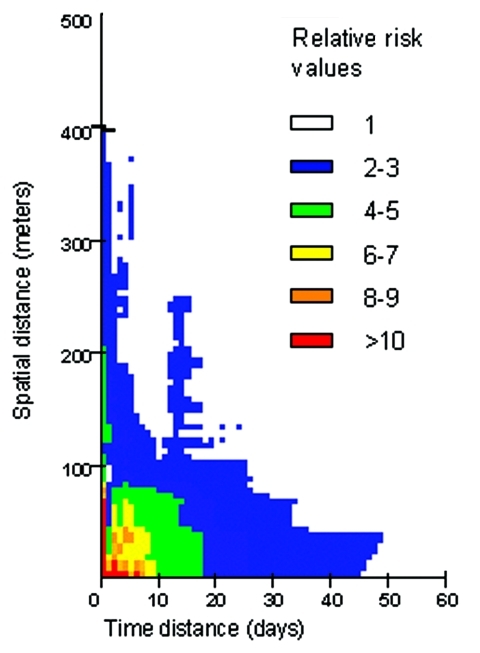
Global representation of the relative-risk (RR) calculated from the confirmed cases data, when space-distance and time-distance from a first theoretical dengue case vary respectively from 0 to 500 m and from 0 to 60 days. Color indicates RR values greater than one (p < 0.001). High RR values are in red.

A first analysis of the RR variations when space and time distances vary over all the epidemic’s extent highlights a main risk area, with RR > 1 (p < 0.001) (Figure 4). This area corresponds to a substantial increase in the theoretical risk of the occurrence of another dengue case. This area is active inside the boundaries of 400 m and 40 days.

**Figure 5:**
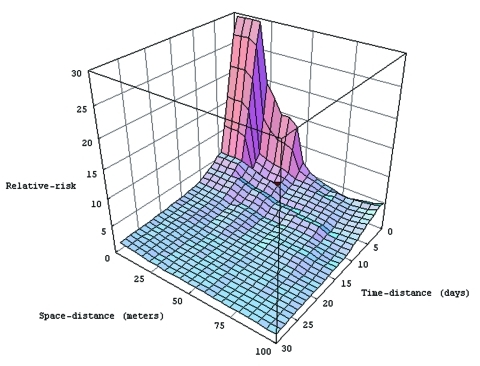
Three-dimensional representation of the main risk area for dengue fever (within 100 m and 30 days’ boundaries) derived from data on confirmed cases.

**Figure 6:**
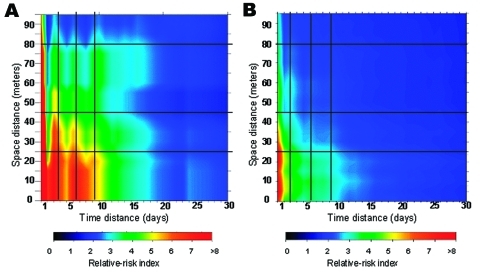
Main risk area for dengue fever (within 100 m and 30 days’ boundaries), derived from laboratory-positive cases data (a) and all suspected cases data (b). Vertical dark lines indicate an apparent temporal periodicity, and horizontal dark lines correspond to apparent spatial breaks.

A more detailed analysis of this risk area shows a strong heterogeneity: an area is very high risk (RR > 5) at short distances (15 m) and over brief periods (6 days). Beyond these space-time limits, the RR rapidly decreases (Figure 5). Moreover, particular patterns are observed, like a temporal periodicity, with peaks of risk every 3 days (Figure 6a). Spatial breaks seem to appear at the approximate distances 20–25 m, 45–50 m, and 80–85 m (Figure 5 and Figure 6a), showing three different risk levels (Figure 5).

A strong concordance exists between the results obtained by using the dengue laboratory-positive cases and those obtained by using all suspected cases: the space and time boundaries are roughly the same (Figure 6b). Although the RR values are different for the same space and time distances, they are correlated with a high correlation coefficient (r = 0.93; p < 0.05).

## Discussion

To study the dynamic of a DF outbreak in the small municipality of Iracoubo during 2001, we located all patients in space by determining their home address and in time by obtaining the date of onset of symptoms. Although the definition of time-location is obvious, the definition of space-location can be questioned. Indeed, using this factor implies that patients have contracted the disease at home, which is a strong hypothesis. This hypothesis is based on practical constraints (since the residential address is the easiest way to implement a location criterion), and on the results of several studies confirming that dengue risk exposure is more important at home because female *Aedes aegypti* mosquitoes are endophilic and take their blood meal during the day with often a peak in the early morning and in the evening (36), and even sometimes during the night (*37**,**38*).

The difficulty of locating each patient’s home has to be pointed out, however. Previous studies had to face the major problem of locating each address and verifying it in the field, which requires a substantial time investment (*4*). For our study in Iracoubo, the relatively small group of patients was easily and quickly located by using maps and aerial photographs. Nevertheless, in the context of an operational dengue surveillance system deployment, our alternative to address georeferencing is not adapted. Therefore, an original interactive software for georeferencing cases by using aerial photographs and maps, during the consultation by the physician or in healthcare centers, was implemented in French Guiana (DOC_teur Software) (*39*). This could be an alternative solution for the problem of georeferencing cases, provided that healthcare centers have computer capabilities.

An initial interpretation of the spatial dengue distribution shows that all areas of the municipality were rapidly affected by the disease. Moreover, the distribution highlights spatial case-clusters inside individual houses and in the nearby neighborhoods of case-patients (Figure 2). One of the aims of the spatial and temporal patterns analysis was to clarify this qualitative interpretation.

Our study on space-time patterning led us to map in space and time the RR for DF within a particular space-time window from the first hypothetical suspected case. This RR index map allowed us to determine the boundaries in space and time of the maximum dengue transmission focus extent (400 m, 40 days) and to identify a very high-risk area at a short distance (15 m) over a short period (6 days). These results confirm the focal nature of DF as reported in the literature, and, above all, fix quantitative values for the transmission focus limits.

Moreover, the strong heterogeneity apparent in the RR index map (Figure 6) is coherent with known entomologic and epidemiologic factors. Indeed, the marked 3 days periodicity is consistent with the length of the gonotrophic cycle of the female *A. aegypti* mosquito (*36*). After being fed and achieving extrinsic incubation, a mosquito bite would be infectious and lead to a human dengue case after the intrinsic incubation period; whether the mosquito bites every 3 days and whether we assumed that intrinsic incubation period is constant in duration, then other dengue cases would be appear every 3 days.

On the other hand, spatial breaks in the disease occurrence seem to correspond roughly to the spatial distances between houses as determined with aerial photographs. Indeed, aerial photo-interpretation shows that for each house, the direct neighboring house is included, in average, in a 25-m radius, which also includes the risk area shown by our results for dengue occurrence. The two next distance peaks, namely 45 m and 80 m, correspond to the third and fourth nearest areas of housing, respectively.

Those similarities between patterns in the RR map derived from space-time location of dengue cases and known transmission factors confirm the relevance of using GIS for the epidemic description. In particular, the available data seem consistent with the hypothesis that most people were infected at home or near the home during the Iracoubo epidemic.

In future studies, obtaining the exact incidence in the exposed population will be preferable. For this goal, a prospective seroepidemiologic study must be conducted in the overall exposed population to identify all dengue cases, including the asymptomatic cases. This kind of study would certainly increase the accuracy of the GIS for the epidemic description. In Iracoubo the distribution of the nonsymptomatic cases and the nonreported cases likely paralleled the spatial distribution of the reported cases. Thus, the fact that we did not dispose of the total number of dengue cases induced more likely a decrease in the precision, than an inaccurate representation of dengue transmission. This hypothesis will be tested in a future study.

These first results show that an objective description of a dengue virus spread using GIS and space-time statistics allows epidemiologists to define risk areas and risk periods, which are necessary for implementing an efficient surveillance strategy. Moreover, the strong concordance of the two RR maps derived from the confirmed cases and suspected cases indicates that a surveillance program could be based on information concerning all suspected cases. Including such information would allow a better response to an outbreak.

Analyzing RR representation shows a very high risk area 6 days after and at <15 m from a first hypothetical dengue case (Figure 5). Because of the short duration of the dengue intrinsic incubation period, each dengue patient contracted the disease a few days before its clinical expression. As a consequence, there were no means of reducing the first RR peak after the first dengue case was detected. Nevertheless, vector control could have reduced the secondary RR peaks, which occurred 3, 6, and 9 days after the first high RR area was identified (Figure 6).

These results could increase the efficiency of the vector-control strategy. Indeed, the RR representation indicates that vector control should be more efficient when conducted inside the houses and against adult mosquitoes. All houses inside a 100-m neighborhood should be treated. The distance of 100 m corresponds to a statistical threshold, which is a result of our study: outside of 100 m around the dengue focus, the probability of observing a dengue case is low. However, if the number of cases reported increases, we will likely increase the precision of such reporting, and this threshold could vary. If these results are confirmed in the future, this would likely lead to improvements in indoor vector control (by indoor spraying of insecticide) during dengue epidemics, in particular in the houses near a house where a confirmed or suspected case occurred, provided that the mosquitoes have been shown to be sensitive to the insecticide. Reducing breeding sites and increasing indoor vector control could be the major means of controlling dengue spread during an epidemic.

## Conclusion

The use of a GIS in a dengue surveillance program requires an efficient case location system and a concerted effort by all health stakeholders: physicians, hospitals, pathology laboratories, and vector control agencies. In French Guiana, a research program named S2Dengue (Spatial Surveillance of Dengue) joins the different health stakeholders for the real-time collection of all dengue-related information (suspected and confirmed cases, vector densities, etc.). The first objective of this project is to provide all participants with weekly maps of dengue incidence to improve prevention measures. The second objective is to link this information with relevant environmental factors and establish a model of the epidemic dynamic.

This program will allow us to validate our results concerning the characteristics of the dynamic of dengue in French Guiana and confirm the potential of using geographic information systems for dengue surveillance at a country level. This effort will also contribute to dengue control strategy.

## References

[1] Gatrell AC, Löytönen M. GIS and health research. An introduction. In: Gatrell AC, Löytönen M, editors. GIS and health: GISDATA 6. Washingtion: Taylor and Francis, Inc; 1998. p. 3–16.

[2] Chaput EK, Meek JI, Heimer R. Spatial analysis of Human Granulocytic Ehrlichiosis near Lyme, Connecticut. Emerg Infect Dis. 2002;8:943–8. 12194771 10.3201/eid0809.020103 PMC2732548

[3] Chadee DD, Kitron U. Spatial and temporal patterns of imported malaria cases and local transmission in Trinidad. Am J Trop Med Hyg. 1999;61:513–7. 10548282 10.4269/ajtmh.1999.61.513

[4] Morrison AC, Getis A, Santiago M, Rigau-Perez JG, Reiter P. Exploratory space-time analysis of reported dengue cases during an outbreak in Florida, Puerto Rico, 1991–1992. Am J Trop Med Hyg. 1998;58:287–8. 9546405 10.4269/ajtmh.1998.58.287

[5] Tran A, Gardon J, Weber S, Polidori L. Mapping disease incidence in suburban areas using remotely sensed data. Am J Epidemiol. 2002;156:662–8. 10.1093/aje/kwf091 12244035

[6] Boone JD, McGwire KC, Otteson EW, DeBaca RS, Kuhn EA, Villard P, Remote sensing and geographic information systems: charting Sin Nombre virus infections in deer mice. Emerg Infect Dis. 2000;6:248–58. 10.3201/eid0603.000304 10827114 PMC2640872

[7] Bavia ME, Hale LF, Malone JB, Braud DH, Shane SM. Geographic Information Systems and the environmental risk of schistosomiasis in Bahia, Brazil. Am J Trop Med Hyg. 1999;60:566–72. 10348229 10.4269/ajtmh.1999.60.566

[8] Beck LR, Rodriguez MH, Dister SW, Rodriguez AD, Rejmankova E, Ulloa A, Remote Sensing as a landscape epidemiologic tool to identify villages at high risk for malaria transmission. Am J Trop Med Hyg. 1994;51:271–80. 7943544 10.4269/ajtmh.1994.51.271

[9] Robinson TP. Geographic Information Systems and the selection of priority areas for control of tsetse-transmitted trypanosomiasis in Africa. Parasitol Today. 1998;14:457–60. 10.1016/S0169-4758(98)01336-2 17040848

[10] Hightower AW, Ombok M, Otieno R, Odhiambno R, Oloo AJ, Lal AA, A geographic information system applied to a malaria field study in western Kenya. Am J Trop Med Hyg. 1998;58:266–72. 9546401 10.4269/ajtmh.1998.58.266

[11] Franck C, Fix AD, Peña CA, Strickland GT. Mapping Lyme disease incidence for diagnostic and preventive decisions, Maryland. Emerg Infect Dis. 2002;8:427–9. 10.3201/eid0804.000413 11971779 PMC2730243

[12] Guerra M, Walker E, Jones C, Paskewitz S, Cortinas MR, Stancil A, Predicting the risk of Lyme disease: habitat suitability for *Ixodes scapularis* in the North Central United States. Emerg Infect Dis. 2002;8:289–96. 10.3201/eid0803.010166 11927027 PMC2732460

[13] Dister SW, Fish D, Bros SM, Frank DH, Wood BL. Landscape characterization of peridomestic risk for Lyme disease using satellite imagery. Am J Trop Med Hyg. 1997;57:687–92. 9430528 10.4269/ajtmh.1997.57.687

[14] Gubler DJ. Dengue. In: Monath TP, editor. Volume 2: the arboviruses: epidemiology and ecology. Boca Raton (FL): CRC; 1988. p. 231–61.

[15] Deparis X, Roche C, Murgue B, Chungue E. Possible dengue sequential infection: dengue spread in a neighbourhood during the 1996/97 dengue-2 epidemic in French Polynesia. Trop Med Int Health. 1998;3:866–71. 10.1046/j.1365-3156.1998.00330.x 9855397

[16] Reiskind MH, Baisley KJ, Calampa C, Sharp TW, Watts DM. Epidemiological and ecological characteristics of past dengue virus infection in Santa Clara, Peru. Trop Med Int Health. 2001;6:212–8. 10.1046/j.1365-3156.2001.00703.x 11299038

[17] Carbajo AE, Schweigmann N, Curto SI, de Garín A, Bejarán R. Dengue transmission risk maps of Argentina. Trop Med Int Health. 2001;6:170–83. 10.1046/j.1365-3156.2001.00693.x 11299033

[18] Koopmans JS, Prevot SR, Vaca Marin MA, Gomez Dantes H, Zarate Aquino ML, Longini IM, Determinants and predictors of dengue infection in Mexico. Am J Epidemiol. 1991;133:1168–78. 2035520 10.1093/oxfordjournals.aje.a115829

[19] Waterman SH, Novak RJ, Sather GE, Bailey RE, Rios I. Gubler DJ. Dengue transmission in two Puerto Rican communities in 1982. Am J Trop Med Hyg. 1985;34:625–32. 4003671 10.4269/ajtmh.1985.34.625

[20] Halstead SB, Scanlon JE, Umpaivit P, Udomsaki S. Dengue and Chikungunya virus infection in man in Thailand, 1962–1964. Part IV: epidemiologic studies in the Bangkok metropolitan area. Am J Trop Med Hyg. 1969;18:997–1021. 4390977 10.4269/ajtmh.1969.18.997

[21] Russell PK, Quy DV, Nisalak A, Simasathien P, Yuill TM, Gould DJ. Mosquito vectors of dengue viruses in South Vietnam. Am J Trop Med Hyg. 1969;18:455–9. 5768779 10.4269/ajtmh.1969.18.455

[22] Ventura AK, Hewitt CM. Recovery of dengue-2 and dengue-3 viruses from man in Jamaica. Am J Trop Med Hyg. 1970;19:712–5. 4987551 10.4269/ajtmh.1970.19.712

[23] Kuno G. Review of the factors modulating dengue transmission. Epidemiol Rev. 1995;17:321–35. 8654514 10.1093/oxfordjournals.epirev.a036196

[24] Deparis X, Murgue B, Roche C, Cassar O, Chungue E. Changing clinical and biological manifestations of dengue during the dengue-2 epidemic in French Polynesia in 1996/97. Description and analysis in a prospective study. Trop Med Int Health. 1998;3:859–65. 10.1046/j.1365-3156.1998.00319.x 9855396

[25] Fouque F, Reynes JM, Moreau JP. Dengue in French Guiana, 1965–1993. Bull Pan Am Health Organ. 1995;29:147–55. 7640693

[26] Institut National de la Statistique et des Etudes Economiques. Recensement de la population française, mars 1999. Exploitation principale (CD-ROM). Paris, France.

[27] Centers for Disease Control. Case definition for public health surveillance. MMWR Morb Mortal Wkly Rep. 1990;39:10–1.

[28] Bundo K, Igarashi A. Antibody-capture ELISA for detection of immunoglobulin M in sera from Japanese encephalitis and dengue hemorrhagic fever patients. J Virol Methods. 1985;11:15–22. 10.1016/0166-0934(85)90120-X 2989309

[29] Clarke DH, Casals J. Techniques for hemagglutination and hemagglutianation-inhibition with arthropod-borne viruses. Am J Trop Med Hyg. 1958;7:561–73. 13571577 10.4269/ajtmh.1958.7.561

[30] World Health Organization. Dengue haemorrhagic fever: diagnosis, treatment, and control. 2nd ed. Geneva: The Organization; 1997.

[31] Reynes JM, Laurent A, Deubel V, Telliam E, Moreau JP. The first epidemic of dengue hemorrhagic fever in French Guiana. Am J Trop Med Hyg. 1994;51:545–53. 7985746

[32] Lanciotti RS, Calisher CH, Gubler DJ, Chang GJ, Vorndam AV. Rapid detection and typing of dengue viruses from clinical samples by using reverse transcriptase-polymerase chain reaction method. J Clin Microbiol. 1992;30:545–51. 1372617 10.1128/jcm.30.3.545-551.1992 PMC265106

[33] GeoConcept, version 5.0. Manuel d’utilisation. GeoConcept SA, Paris, France, 2000.

[34] Knox EG. The detection of space-time interactions. Appl Stat. 1964;13:25–9. 10.2307/2985220

[35] Surfer, version 7.02. Golden (CO): Golden Software, Inc, 2000.

[36] Rodhain F. L’écologie *d’Aedes aegypti* en Afrique et en Asie. Bull Soc Pathol Exot. 1996;89:103–6. 8924766

[37] Chadee DD, Marting R. Landing periodicity of *Aedes aegypti* with implications for dengue transmission in Trinidad, West Indies. J Vector Ecol. 2000;25:158–63. 11217215

[38] Diarrassouba S, Dossou-Yovo J. Rythme d’activité atypique chez *Aedes aegypti* en zone de savane sub-soudanienne de Côte d’Ivoire. Bull Soc Pathol Exot. 1997;90:361–3. 9507772

[39] Bouix A. S2E DENGUE, surveillance spatiale de la Dengue et son interface avec le logiciel médical DOC_teur…ou comment commencer avec un Système d'Information Géographique! Manuel d’utilisation du logiciel DOC_teur, Kourou, French Guiana, 2003.

